# Investigation of Fatigability during Repetitive Robot-Mediated Arm Training in People with Multiple Sclerosis

**DOI:** 10.1371/journal.pone.0133729

**Published:** 2015-07-27

**Authors:** Deborah Severijns, Johanna Renny Octavia, Lore Kerkhofs, Karin Coninx, Ilse Lamers, Peter Feys

**Affiliations:** 1 REVAL Rehabilitation Research Centre, BIOMED, Faculty of Medicine and Life Sciences, Hasselt University, Diepenbeek, Belgium; 2 Parahyangan Catholic University, Industrial Engineering Department, Ciumbuleuit 94, Bandung, 40141, Indonesia; 3 Hasselt University—tUL–iMinds, Expertise Centre for Digital Media, Diepenbeek, Belgium; 4 Rehabilitation and MS centre Overpelt, Overpelt, Belgium; Kessler Foundation Research Center, UNITED STATES

## Abstract

**Background:**

People with multiple sclerosis (MS) are encouraged to engage in exercise programs but an increased experience of fatigue may impede sustained participation in training sessions. A high number of movements is, however, needed for obtaining optimal improvements after rehabilitation.

**Methods:**

This cross-sectional study investigated whether people with MS show abnormal fatigability during a robot-mediated upper limb movement trial. Sixteen people with MS and sixteen healthy controls performed five times three minutes of repetitive shoulder anteflexion movements. Movement performance, maximal strength, subjective upper limb fatigue and surface electromyography (median frequency and root mean square of the amplitude of the electromyography (EMG) signal of the anterior deltoid) were recorded during or in-between these exercises. After fifteen minutes of rest, one extra movement bout was performed to investigate how rest influences performance.

**Results:**

A fifteen minutes upper limb movement protocol increased the perceived upper limb fatigue and induced muscle fatigue, given a decline in maximal anteflexion strength and changes of both the amplitude and the median frequency of EMG the anterior deltoid. In contrast, performance during the 3 minutes of anteflexion movements did not decline. There was no relation between changes in subjective fatigue and the changes in the amplitude and the median frequency of the anterior deltoid muscle, however, there was a correlation between the changes in subjective fatigue and changes in strength in people with MS. People with MS with upper limb weakness report more fatigue due to the repetitive movements, than people with MS with normal upper limb strength, who are comparable to healthy controls. The weak group could, however, keep up performance during the 15 minutes of repetitive movements.

**Discussion and Conclusion:**

Albeit a protocol of repetitive shoulder anteflexion movements did not elicit a performance decline, fatigue feelings clearly increased in both healthy controls and people with MS, with the largest increase in people with MS with upper limb weakness. Objective fatigability was present in both groups with a decline in the muscle strength and increase of muscle fatigue, shown by changes in the EMG parameters. However, although weak people with multiple sclerosis experienced more fatigue, the objective signs of fatigability were less obvious in weak people with MS, perhaps because this subgroup has central limiting factors, which influence performance from the start of the movements.

## Introduction

Multiple sclerosis (MS) is a chronic auto-immune disease, causing sensory as well as motor problems, such as muscle weakness, tremor and spasticity leading to limitations in walking and functional mobility, but also upper limb dysfunction [1,2].

People with MS (PwMS) benefit from resistance and endurance training which was shown to improve muscle strength, physical fitness, functional mobility and quality of life [3]. It is well-known in neurorehabilitation that both training intensity and volume are important to achieve optimal strength gains and functional recovery [4]. In upper limb rehabilitation, robot-mediated therapy has the potential to facilitate a high number of repetitions leading to neurorehabilitative effects [4,5]. Pilot studies have already showed the benefits of repetitive arm training by the use of robotic therapy, both in stroke patients and in PwMS [6–8]. Clinically, it is, however, seen that PwMS need to limit their exercise duration due to excessive fatigue when performing repeated muscle exercises. Qualitative research also revealed that some patients complained about muscle fatigue and pain, the latter perhaps related to compensatory trunk and shoulder girdle movements to keep performance up despite motor fatigue [9]. Kluger et al. [10] propose to use the term fatigability to refer to objective changes in performance relative to a criterion or reference value, and fatigue to indicate subjective sensations. Fatigability during motor tasks is also referred to as motor or muscle fatigue and can be described as [11], “an exercise–induced reduction in the ability of muscles to produce force or power, regardless of whether a task can be sustained”. Fatigability can be assessed with a decline in force [11]. Surface electromyography (sEMG) has also been used to detect objective signs of muscle fatigue, underlying fatigability [12]. Both frequency and amplitude parameters are indicative for muscle fatigue [13]. The clinical value of these fatigue related changes in the sEMG signal, has been shown by relating these parameters to the subjective experience of muscle fatigue, for example in the upper trapezius [14].

Excessive fatigability in both lower limbs and upper limbs, as documented with fatigue indices, is present in a subset of PwMS [15–17]. The occurrence of motor fatigue during walking has been documented [18], but no reports have been published showing this excessive fatigability during exercise sessions of the upper limbs.

Recently, Octavia et al.[19] performed a pilot study in six PwMS to investigate the occurrence of muscle fatigue during 15 minutes of robot-mediated game exercise, requiring physical and cognitive efforts. Observations suggested that muscle fatigue development was associated with a decline in game performance. The present cross-sectional controlled study expands previous pilot investigation of exercise-related upper limb fatigability, but in a larger sample and with better controlled physical efforts. In short, this study investigated whether abnormal fatigability was present, measured by subjective ratings of upper limb fatigue, maximal force tasks, sEMG and performance.

Subjects performed repetitive shoulder anteflexion movements in a game-like virtual environment during five movement bouts of three minutes. During or in-between this robot-mediated movement, performance measures, surface sEMG and subjective experience of fatigue were recorded. Evaluations were repeated after 15 minutes of rest. We hypothesized that PwMS subjectively experience more fatigue than healthy controls and that they show a higher fatigability with a larger decline in performance measures over time which would show in sEMG parameters.

## Methods

### Subjects and descriptive measures

PwMS were recruited in the MS and Rehabilitation Centre, Overpelt. Inclusion criteria for the PwMS were: a definite clinical diagnosis of MS according to the McDonald criteria, age above 18. The PwMS were excluded when they were treated with glucocorticosteroid treatment one month prior to participation, had very severe upper limb paralysis (MI<18/100) impeding goal-directed arm movements and other cognitive or visual impairments hindering navigating with the robot in a virtual learning environment. Six PwMS simultaneously also participated in an intervention study within the Interreg IV project entitled “Rehabilitation Robotics II” (IVA-VLANED-1.58) [9]. Healthy controls of comparable age and sex were recruited from family and friends. Participants with known shoulder pain or orthopaedic problems were excluded. The presented study was approved by the Ethical committee of the Universitaire ziekenhuizen K.U.Leuven, Universiteit Hasselt and Mariaziekenhuis Overpelt. All participants gave written informed consent prior to inclusion in the study.

The generic descriptive measures were age, gender, hand preference defined by means of the Oldfield handedness questionnaire. All participants were asked to fill out a fatigue questionnaire, the Dutch Modified Fatigue Impact Scale (MFIS) [20] to describe the impact of fatigue on daily life. For the PwMS, the Expanded Disability Status Scale (EDSS), years since diagnosis, and MS phenotype were collected. To describe the clinical characteristics of the upper limb capacity of the PwMS, the Action Research Arm test (ARAT) was performed while the muscle strength of the tested arm was measured with the Jamar hand grip module and the Motricity Index of the upper limb [21]. In the healthy controls, these clinical descriptive outcome measures of muscle strength and arm function were not recorded, since we assumed that included controls without problems with upper limb functions, would show normal maximal strength.

### Apparatus

We used the Haptic Master (Moog bv., Nieuw-Vennep, the Netherlands) with the I-TRAVLE system. Custom-made hardware and software was used to facilitate repetitive anteflexion movements with the Haptic Master [9,22,23]. The Haptic Master is equipped with a gimbal mounted hand brace, in which the hand is fixed. A 40" monitor was used as a visual display and was placed 1.5 m in front of the patient.

To record muscle activity, a wireless EMG system was used. Bipolar and pre-amplified surface electrodes with a fixed distance of 1.0 cm (Trigno wireless system, Delsys Inc., Natick, MA, USA) were used to record the muscle activity. sEMG was sampled at 2000 Hz, with a bandwidth of 20–450Hz. The Common mode rejection rate was >80dB. Delsys EMG Works software, version 4.0.2 (Delsys, Inc., Natick, MA, USA) was used for signal acquisition. The EMG system was connected to a standard laptop where the EMG files were stored for further analysis. Further data-analysis was done with custom-made software (using Labview, National Instruments Cooperation, Austin, TX).

### Movement protocol

**Fig 1:**
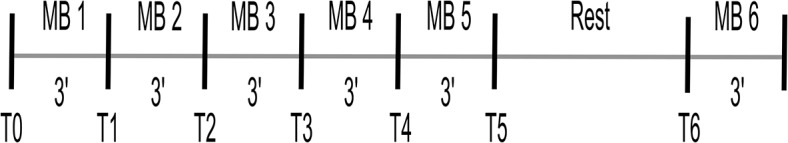
Schematic representation of the experimental design. (MB = Movement bout; T = testmoment; ‘ = minute).

**Fig 2:**
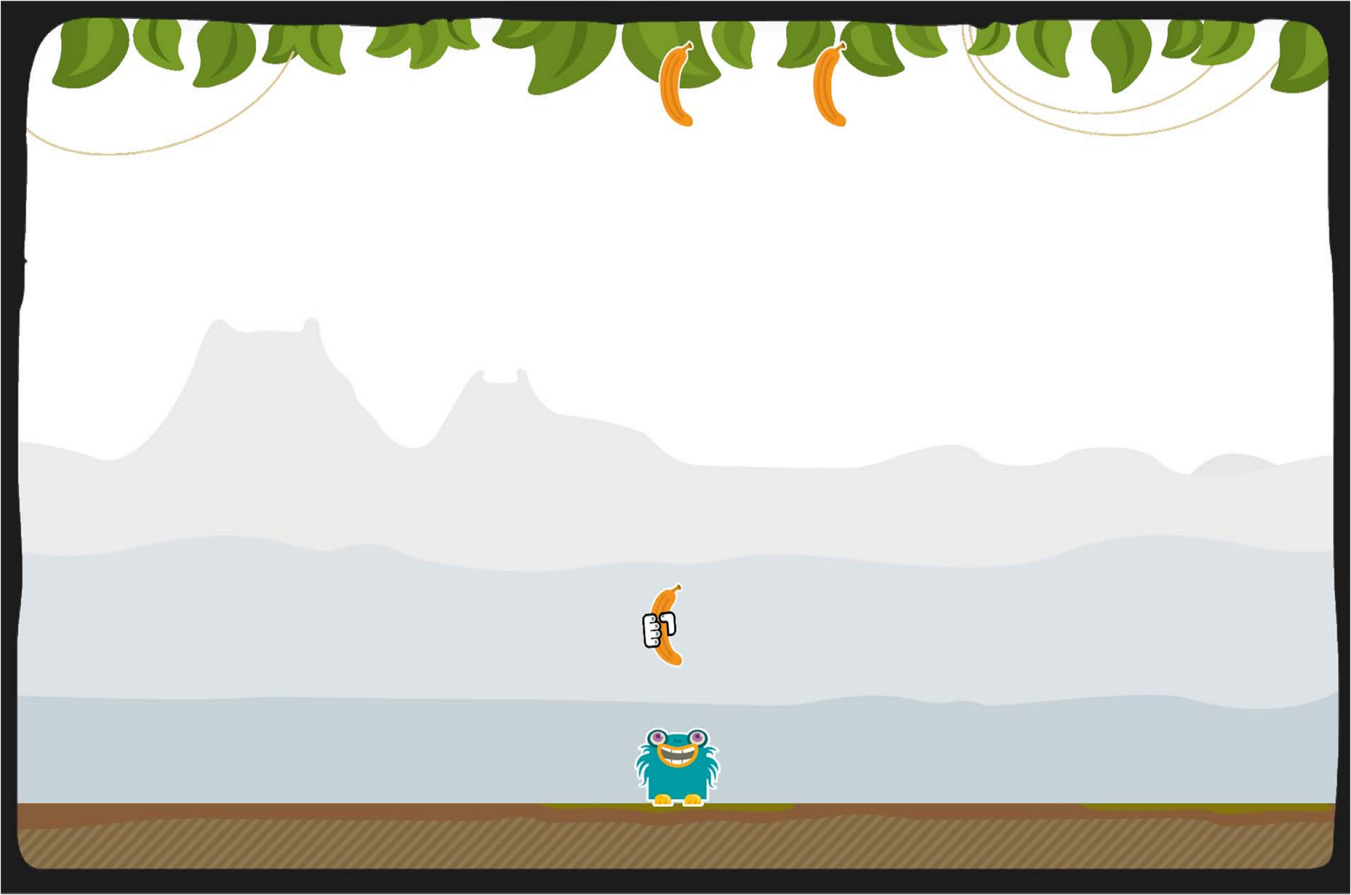
Screenshot of the virtual environment that was created to facilitate repetitive anteflexion movement.

All subjects performed a movement protocol, consisting of 5 bouts of 3 minutes of repetitive anteflexion movements (Fig 1) to 80% of the maximal anteflexion movement range of motion with a light weight of 2.5 N. The anteflexion movements were goal-directed as they had to be accurate in space to pick fruit by stabilising for three seconds on top of the visual display and feed fruit to an avatar at the bottom of the screen in a virtual environment (Fig 2). The rhythm of the anteflexion movements was not externally paced.

Before and after each movement bout (test moments, T0-T7), (sub)maximal contractions were performed and the subjects filled out a Visual Analogue Scale (VAS). After five bouts of anteflexion movements, there was a resting period of 15 minutes, followed by one last bout of three minutes.

### Procedure

Prior to experimental testing, the skin over the shoulder muscles was shaved and wiped with 70% isopropyl alcohol. The sEMG electrodes were placed according to the SENIAM guidelines on the anterior and middle deltoid, the biceps brachii and the upper trapezius muscle [24]. All participants received verbal instructions and were familiarised with the use of the device and the lifting exercise, without performing a complete set of 3 minutes anteflexion, to avoid interference with the following protocol. One hand of the subjects was attached to the gimbal, to control the Haptic Master robot, after which the maximal active range of motion in all directions with the Haptic Master was determined. Hereafter the movement protocol started, as described above.

### Outcome measures assessed in-between the movement bouts

A 30-second isometric contraction in 90° of shoulder anteflexion was asked with visual feedback of the position on the screen (the instruction was to keep a ball between two lines). During this contraction, the sEMG of the anterior deltoid was sampled. These sEMG signals were post-processed to calculate the Root mean square (RMS) and the median frequency (MDF) over the 30 seconds period. This was followed by a maximal voluntary isometric contraction (MVC) in 90° of shoulder anteflexion, where the subjects were asked to push up as hard as they could. The MVC anteflexion in Newton was measured by the haptic master. All subjects were asked to score how fatigued their arm was on a visual analogue scale (VAS), by answering the question: “How fatigued is your arm?”. They had to indicate their upper limb fatigue feelings on a line of 10 cm, where one end (0 cm) represented: not fatigued at all, while the other end (10 cm) represented the answer: the most fatigued as can be. The score was determined with a ruler and noted with one decimal number.

### Performance measures during the movement bouts

The performance of repetitive anteflexion movements during the ‘picking fruit’ task was measured by means of the following measures: the average movement trajectory (metres), average movement time per trajectory (seconds) and the total number of fruit picked (total number of anteflexion movements) during the task.

### Data-analysis

All statistical analyses were performed using SAS JMP Pro 11.2.0 (2013, SAS Institute inc.). Descriptive statistics are shown in mean± standard deviation.

The performance measures during the movement bouts (movement trajectory, movement time per trajectory and number of fruit picked) and the tests between the consecutive movement bouts (VAS, MVC anteflexion and the RMS and MDF from the sEMG) were analysed with a linear mixed model. Time, referring to test moment (T0-T5) or movement bouts (MB1-MB5), and group (healthy controls vs. PwMS) and the group*time interaction effect were included as fixed effects. To accommodate that all observations coming from the same patient are correlated and that there is between-patient variation, a random patient effect was added to the model.

To document if there was a change in the parameters after the rest period, a mixed model was used to compare T5 with T6 and MB5 with MB6 with a possible group*time interaction effect.

Pairwise comparisons were performed with a Tukey HSD test, where, in each group separately, consecutive tests or movement bouts were compared with each other. This test includes a Tukey-Kramer correction for multiple testing.

A correlation analysis between subjective and objective measures was performed. To this end, delta scores were calculated between T0 and T5 for the VAS score, the MVC anteflexion and the RMS and MDF of the prime mover, the anterior deltoid. The correlation between the general fatigue questionnaire and the delta scores of the VAS was also calculated. For all correlations, Spearman correlation coefficients were used.

To further analyse if increased fatigability is due to the presence of the disease of MS or the presence of muscle weakness, a subgroup-analysis was performed between a subgroup of PwMS with hand grip muscle weakness, a subgroup of PwMS with normal hand grip strength in comparison with a healthy control group. Weak hand grip strength was defined as a maximal hand grip strength below age-and gender-comparable norms [25].

The significance level for all analyses is set to 5%.

## Results

### Subject characteristics

Sixteen PwMS (55± 8 yrs) and healthy controls (54± 10 yrs) participated in this study. An overview of the clinical characteristics are presented in Table 1. For all participants, except for two PwMS, the dominant hand was tested resulting in 5/16 of the PwMS being tested with the left arm. The right arm was tested in the healthy subjects. Ten of the sixteen PwMS reported to be fatigued during the performance of daily life activities as indicated by the total score on the MFIS, while none of the healthy controls was fatigued according to the cut-off value of 38 [26]. Eight out of sixteen PwMS showed hand grip weakness, when compared with normative age and sex-matched data[25].

**Table 1:** Subject characteristics.

	HC (n = 16)	PwMS (n = 16)
**Sex (M/F)**	5/11	6/10
**Age (years)**	54±10	55±8
**Hand Dominance (R/L)**	16	13/3
**Type of MS (RR/SP/PP)**	n.a.	3/10/3
**Disease duration (years)**	n.a.	15±9
**EDSS (median, range)**	n.a.	6 (2–8)
**MFIS (0–88)**	16.3±13.1	42.3±17.8
**Motricity index (0–100)**	n.a.	76.4±14.0
**ARAT (0–57)**	n.a.	45.9±14.2
**Hand grip strength (kg)**	n.a.	21.6±10.2

RR: relapsing-remitting MS; SP: Secondary progressive MS; PP: Primary progressive MS; EDSS: Expanded Disability Status Scale; ARAT: action research arm test, MFIS: Modified Fatigue Impact Scale. All measures were recorded on the tested arm.

### Outcome measures assessed in-between the movement bouts

**Fig 3:**
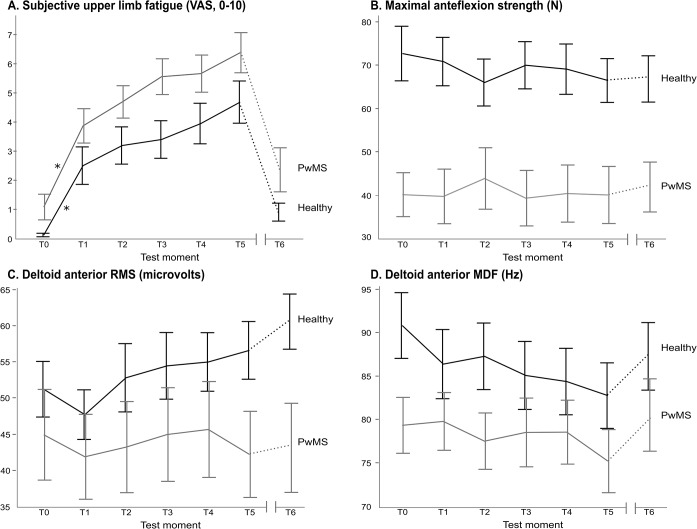
Means and standard errors of the outcome measures, assessed in-between the movement bouts. (A) Subjective fatigue of the upper limb (0–10). (B) Maximal anteflexion strength (N). (C) Amplitude of the EMG signal of the anterior deltoid during 30 seconds isometric shoulder anteflexion in 90°. (D) Median frequency of the EMG signal of the anterior deltoid during 30 seconds isometric shoulder anteflexion in 90°. PwMS: people with multiple sclerosis. T0: test moment before the first movement bout. T1-T5: test moment after 3 minutes of anteflexion movements. * represents a significant difference between the consecutive test moments. The interruption of the x-axis represents the resting period of 15 minutes, where after T6 is measured. This is represented on the graph by a dashed line.

An overview of the results on the different outcome measures is shown in Fig 3. The average group data are shown in S1 Table. The scores on the question “is your arm fatigued?” were not significantly higher in PwMS, compared to the healthy controls (Fig 3A). There was a significant main effect of time (p<0.0001), but no significant group*time interaction effect, which indicates no difference in the increase of the perceived upper limb fatigue between both groups. The largest increase in fatigue feelings is seen between T0 and T1, where the only significant pairwise comparison was found (p< 0.0001 for both groups). To investigate the changes after a rest period of 15 minutes, T5 was compared with T6. After rest, the VAS score decreased in both groups (main effect of time; p<0.001), without a group*time interaction effect.

The MVC anteflexion was significantly higher in healthy controls than in PwMS (main effect of group: p<0.001), while the strength declined over time (p<0.05) in both groups (Fig 3B). Although the interaction was not significant, it should be noted that the PwMS had on average less decline in MVC anteflexion, compared to the healthy controls after the 5 movement bouts (-2.57N vs. -6.22N). After rest, the strength did not increase significantly in either group. PwMS still had a lower MVC anteflexion than healthy controls (p<0.05), without an interaction effect of group*time.

To assess if muscle fatigue developed during the short movement bouts, RMS values and MDF values of the anterior deltoid muscle were calculated (Fig 3C and 3D). The RMS values during a 30 seconds isometric contraction after each exercise bout showed a significant main effect of time (p<0.01), but no main effect of group. There was an interaction effect of group*time (p<0.01), with a higher increase of RMS in healthy controls, compared to PwMS (Fig 3C). The MDF decreased over time (p<0.0001) and showed an interaction effect between group and time (p<0.05), with a steeper decrease in healthy controls (Fig 3D). On group level, an average decline of 8% and 5.4% of the MDF during the movement bouts was found in the healthy controls and PwMS, respectively. To investigate if the sEMG parameters changed after rest, T5 was compared with T6. After rest, the RMS did not show a main effect of group, time or a group*time interaction effect. The MDF of the EMG signal had no group effect or a group*time interaction effect, but there was a time effect with p<0.0001, indicating that MDF of the anterior deltoid increased over time.

To analyse if the changes in subjective perception of the fatigue of the arm were correlated to the objective fatigue parameters, derived from the sEMG signal, the correlation coefficient was calculated between the difference scores (T5-T0) of the VAS score and the RMS and MDF of the anterior deltoid. There was no significant correlation between the changes in the objective EMG parameters and subjective fatigue feelings. No correlation between the scores on the MFIS and the change in subjective fatigue was found in either group. The increase in subjective fatigue was correlated with the changes in MVC anteflexion in PwMS (r = -0.51, p<0.05), but not in healthy controls.

To investigate if increased fatigability is related to the presence of MS or the presence of muscle weakness, a subgroup analysis was performed between three groups (PwMS with muscle weakness, PwMS with normal strength and healthy controls). Results are graphically shown in S1 Fig and an overview of this subgroup analysis in shown in S3 Table.

The PwMS with normal hand grip strength (n = 8/16), were not significantly weaker for the MVC anteflexion, compared to the healthy controls. The healthy controls were stronger on the MVC anteflexion, compared to the PwMS with hand grip weakness (group effect: p<0.001). There was no significant time or group*time interaction effect for the MVC anteflexion.

The PwMS with impaired maximal hand grip strength, showed a more steeper increase in fatigue feelings, compared to the PwMS with normal hand strength and compared to the healthy controls (interaction effect of group*time with p<0.001). Healthy controls and PwMS with normal hand grip strength did not differ in the increase of fatigue feelings in the arm (illustrated in S1 Fig). After rest, all groups showed an equal decrease in perceived fatigue of the upper limb. For the RMS of the anterior deltoid, there was no difference between groups, without any main effect of time. There was a significant group*time interaction effect, but this overall group*interaction effect was not retained when the groups were compared separately. Although there was no group effect for the MDF of the anterior deltoid, there was a significant decrease over time with a group*time interaction effect, where PwMS with normal strength, showed a similar decline in MDF, compared to healthy controls, where the latter groups showed a larger decrease than PwMS with hand grip weakness. After rest, this MDF increased equally for both groups.

### Performance measures during the movement bouts

**Fig 4:**
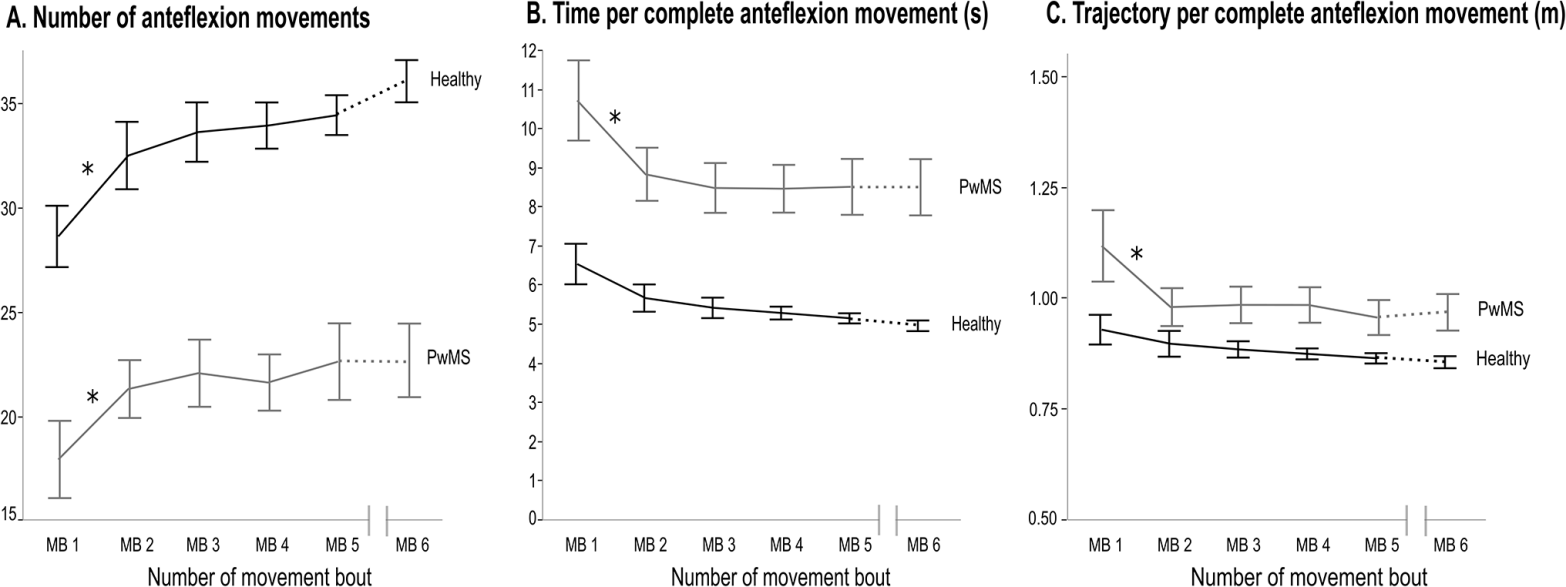
Means and standard errors for the performance measures during the movement bouts of three minutes. (A) number of anteflexion movements; (B) average time of all anteflexion movements; (C) average trajectory of all anteflexion movements. MB: movement bout with a duration of three minutes. * represents a significant difference between the consecutive movement bouts. The interruption of the x-axis represents the resting period of 15 minutes, where after MB 6 is completed. This resting period is represented on the graph by a dashed line.

All movement parameters (movement time, movement trajectory, number of anteflexion movements), showed a significant main effect of time and group (p<0.01). The average group data are shown in S2 Table. Although PwMS did not perform as much movements, both PwMS and healthy controls showed an increase in the number of anteflexion movements and a decrease of the movement time and movement trajectory over time, as shown in Fig 4. According to pairwise comparisons in each group, these changes were only significant between MB1 and MB2 for number of anteflexion movements, movement trajectory and movement time in the PwMS and number of anteflexion movements in healthy subjects. The PwMS scored significantly lower on the number of anteflexion movements (Fig 4A), and accordingly significantly higher on the movement time and movement trajectory (Fig 4B and 4C). No interaction effect was found. After rest, the number of anteflexion movements significantly increased (p<0.05). The increase was higher in healthy controls than in PwMS, shown by a significant interaction effect (p<0.05). There was no significant change after rest for the movement time per cycle, or for the distance travelled.

The subgroup analysis where PwMS with normal hand grip strength, PwMS with hand grip weakness and healthy controls were compared, showed that the number of anteflexion movements, the movement trajectory and the average time needed to perform one anteflexion movement, improved significantly over time (p<0.05) in all groups, with a non-significant group*time interaction effect. There were significant differences between groups, as healthy controls performed better than each MS group. The performance of the PwMS with and without hand grip muscle weakness was not significantly different.

## Discussion

We investigated if PwMS and healthy controls were fatigable during a movement protocol of fifteen minutes, and if so, whether recovery would be present after a period of fifteen minutes of rest.

The anteflexion movements were fatiguing for both groups, as shown by an increase in subjective fatigue throughout the protocol. The maximal anteflexion strength decreased after 15 minutes of repetitive anteflexion movements. The EMG parameters revealed the presence of muscle fatigue related to the anteflexion movements, by showing a significant decline in MDF and a significant increase of RMS of the sEMG of the anterior deltoid muscle during an isometric test contraction in-between the movements. Although several parameters point out that there was some degree of muscle fatigue in both groups, the performance did not decline over time. The lack of differentiation between groups was surprising, as it was hypothesized that PwMS would experience more fatigue and were faster fatigable than healthy controls.

The participants differed individually in fatigue feelings, where some healthy people did not consider this movement to be fatiguing at all, while some of the PwMS considered the movement to be very exhaustive. To analyse this finding in more detail, two subgroups of 8 PwMS each, were created, based on the maximal hand grip strength. Findings need to be interpreted cautiously, since the number of subject in each group is low. We defined the groups according to the deviation of hand grip strength norms [25]. Although hand grip strength is distal upper limb strength and we assessed the shoulder fatigability, this criterion seems valid, since the MVC in shoulder anteflexion was also significantly different between the groups (S1 Fig, panel B). This confirms previous findings on the high relation of hand grip strength and shoulder muscles strength [27].

Subgroup analysis showed that PwMS with hand grip weakness, experience a larger increase in fatigue compared to PwMS with normal hand grip strength, while the latter group experienced the same amount of increase in fatigue as healthy controls. The higher increase in fatigue feelings could be due to the relatively higher intensity of the movements in PwMS with strength impairments. Although the weight was of low-intensity (2.5N), the relative weight is slightly different. Subgroups moved with their upper limb on a different % of their maximal anteflexion strength, which was 10% in the weak PwMS vs. 6% in the PwMS with normal strength and 4% in the healthy control group. The different perception, might be caused by the relative higher weight which needs a higher brain activation to produce the necessary strength in PwMS with muscle weakness. This higher brain activation, with the secondary effect of causing fatigue feelings [28], may be even intensified by the even more extensive neural damage in weak PwMS.

The PwMS with a higher increase in subjective fatigue showed more decrease in MVC anteflexion, as shown by the correlation between these two outcomes. This points to the fact that strength decline over time is related to the general perception of fatigue in PwMS, as already stated before [29]. However, a decline in MVC anteflexion was found both in HC and PwMS. This decrease in MVC was small in PwMS (-2.55N), who on group level, kept their maximal anteflexion strength quite stable, in contrast to what is expected. However, one explanation could be that, in the healthy population, there is a relation between maximal strength and fatigability, where the stronger subjects show more fatigability. This relation remains contradictory in PwMS [17,30,31]. Also, the changes in amplitude and median frequency are smaller in PwMS, compared to healthy controls. Here, the PwMS with muscle weakness showed less change in MDF than the PwMS without muscle weakness. The latter group showed an equal decrease over time than the healthy controls. We would expect, however, that signs of muscle fatigue were more obvious in the weak PwMS, especially since the lifting weight is the same for all subjects. Moreover, earlier evidence showed that PwMS have more Type IIa muscle fibres at the cost of type I fibres, as shown in the leg muscles [32], which are more fatigable than Type I fibres. This was surprisingly not the case, probably because of the fact that the weight, lifted during the movements, was very light. The weight that was used was above 10% of the MVC in only 3 of the 16 PwMS, so the PwMS exercised on average on 8% of their MVC, while the healthy controls exercised at 4% of their MVC. Another explanation could be that there was a large difference in baseline-performance between the groups. PwMS moved slower and performed less repetitions during the three minutes of movement than healthy controls, without any differences between PwMS with or without muscle weakness. For example in the first bout of movement, they did 18 repetitions, where the healthy controls did 28 repetitions. The rest-work cycle is an important factor to avoid muscle fatigue, where even with minimal breaks, muscle fatigue presents later [33]. Next, it might be that the PwMS are limited due to central activation failure, so that the contribution of peripheral muscle fatigue in the weak group of PwMS is limited in contrast to the central fatigue, as hypothesized previously [34,35]. However, in the EMG signal, central factors and peripheral factors cannot fully be discriminated.

Recovery after rest was seen, where the MDF of the anterior deltoid increased after the 15 minutes of rest. This is in line with previous studies, which showed that the power spectrum of an EMG signal quickly recovers with rest [36]. No change in RMS values was found, which could be explained by the fact that the amplitude parameters are less sensitive to muscle fatigue in comparison to the frequency parameters, as stated before in other muscle groups [37]. The RMS can also pose some interpretation problems, as a raise in RMS can indicate muscle fatigue, but also increased muscle recruitment.

Arm function is important for daily life functionality, especially in PwMS with more severe disability [38]. Muscle fatigue is therefore better avoided in the training of PwMS, since excessive muscle fatigue has possible negative effects on arm function after training. Usually, with the standards strength measures, muscle fatigue is detected only after it occurs. However, for PwMS, it seems important that muscle fatigue is detected before the failure point, given the possible impact on daily life performance. We hypothesized that this might be possible by combining information of subjective measures for fatigue, sEMG parameters for muscle fatigue and performance measures for general fatigability. If the subjective fatigue is related to the objective parameters, one could think that the subjective scores of perceived exertion could replace the objective measures. There was, however, no relation between the change in sEMG parameters and the subjective fatigue feeling (VAS score), so probably, these cannot be interchanged.

Instead of a negative effect on the performance due to muscle fatigue and subjective fatigue, a learning effect could be seen in all groups, where the largest improvement is seen from the first to the second movement bout. However, no feedback about the number of repetitions or pacing of movements, with a motivational aspect, was given. Probably, every subject chose the most optimal pace to sustain the movements. Previous literature in healthy subjects also stated, that a successful performance of submaximal exercise can continue, despite signs of muscle fatigue [39]. The fact that the performance can be sustained, could also be due to compensation of other muscles, as recent studies suggest that people adapt their motor behaviour to fatigue to unload fatigued body regions [40]. Even the PwMS with impaired upper limb strength, could keep on the performance and did not differ from the PwMS with normal strength. When a paced rhythm is used, as in most of the game-like exercises, it will be probably more difficult to sustain the exercise for the PwMS. In previous research on fatiguing protocols for the shoulder, the pace at which muscle contractions would take place is usually set [41].This was deliberately not applied here, since we wanted to examine the occurrence of muscle fatigue during a self-guided exercise trial.

Our results showed that PwMS are able to sustain robot-mediated light-weight movements for fifteen minutes. This contrast the findings of an earlier pilot study performed by Octavia et al. [19], who showed a performance decline in 4 out of 6 tested PwMS after fifteen minutes of game-like exercises in a virtual environment. The contrast could be related to the fact that we used a simple anteflexion movement, a unidimensional movement without any further cognitive effort required, while Octavia et al.[19] applied a game requiring three-dimensional movements as well as adaptation to cognitive distractors.

However, one must be cautious to generalize the findings of this study. The subjects in the two groups were of comparable age and gender. There were, however, differences in hand grip strength and general fatigue feelings. These differences are unavoidable when including a representative group of PwMS, since both hand grip weakness [2] and fatigue feelings [42] are present in a substantial group of PwMS. The differential influence of muscle weakness was investigated with a subgroup analysis where weak PwMS were compared with strong PwMS and healthy controls. The groups were not matched on handedness. This is not likely to cause problems with interpretation of the results, as it was already reported that fatigability does not differ between right-handed and left-handed persons [43] and only 3 out of 16 PwMS were left-handed. Further, although we aimed to test the dominant arm in all subjects, two PwMS performed the tests with their non-dominant hand. This is not thought to importantly influence the fatigability results, since previous reports already stated that there was no difference in fatigability between the dominant and non-dominant hands in PwMS [15] and in the elbow in healthy subjects [43,44]. We only did a short movement trial of fifteen minutes. Despite of this very easy task and introduction beforehand, there was a significant learning effect after the first movement bout. However, this improvement in performance was only significant from the first to the second period of 3 minutes. Hereafter, performance remained stable. This learning effect could be caused by the limited practice beforehand. In this study, there was no complete 3 minutes practise trial, to avoid interference with the test protocol afterwards. A separate trial on another test day to introduce the subjects would probably eliminate this learning effect.

On the methodological side, it is noted that only the strength in the vertical-axis was measured to determine MVC anteflexion, so we could not preclude any measurement error, especially in the PwMS, who showed some problems with movement control.

EMG results also need to be cautiously interpreted, as in literature, only negative shifts in MPF greater than 8% were used to consider the presence of muscle fatigue [45,46]. On group level, this was the case for the healthy controls, but not for the PwMS, where the decline in MDF was on average 5.4%. When using the cut-off of 8% change in MDF to be indicative for muscle fatigue on the individual level, there were 9 out of 16 people in the healthy control group who showed signs of muscle fatigue and 5 out of 16 people in the group of PwMS, whereof 4 in the group with normal strength and only 1 in the group of PwMS with muscle weakness. No measurements of skin impedance were performed, therefore, differential influences of skin impedance on group level cannot be ruled out. However, care was taken to minimize skin impedance, by shaving the skin and wiping it with alcohol before placement of the electrodes.

The task used in this study, was of overall light intensity for both groups, therefore, the conclusions drawn here cannot be fully extrapolated to higher intensity exercises which are usually recommended for muscle building. The question remains if there would have been a difference in fatigability between PwMS and healthy controls when individualized high intensity exercises are used. A light weight is effective enough to improve muscle strength, when enough repetitions are performed [47]. It seems that PwMS cannot perform the same amount of repetitions in a fixed time frame as healthy controls. Since the number of performed repetitions is important [39], PwMS should exercise longer to get the same amount of repetitions, or use a higher training weight.

Then, probably, the use of robot-assistance is necessary, for at least a subgroup of the PwMS with muscle weakness at baseline and fatigability. Future research should be done in those subjects who report to have exercise-related motor fatigue, since we did not select exclusively on this specific subgroup. These persons could benefit from adaptive robot-mediated games [22] as well as from gravity compensation [23] and adapted movement complexity. Further, future studies should also incorporate a similar clinical assessment of the upper limbs in the healthy controls, to be able to compare with the data obtained in the patient group.

## Supporting Information

S1 Fig Graphical results in means and standard errors of the subgroup analysis where people with multiple sclerosis with normal hand grip strength are compared to people with multiple sclerosis with hand grip weakness and to healthy controls. PwMS: people with multiple sclerosis. (A) The score for the perceived fatigue in the arm. (B) The maximal anteflexion strength. (C) The number of anteflexion movements within each movement bout of three minutes. (D) Median frequency of the anterior deltoid.
(TIF)

S1 Table Means and standard deviation per group of the scores on the perceived fatigue in the arm, the maximal anteflexion strength and the root mean square and median frequency of the anterior deltoid. PwMS: people with multiple sclerosis.
(DOCX)

S2 Table Means and standard deviation per group of the performance measures during the movement bouts. PwMS: people with multiple sclerosis.
(DOCX)

S3 Table The results of the mixed model analysis where three groups were compared: healthy controls (n = 16), people with multiple sclerosis with muscle weakness (n = 8) and people with multiple sclerosis without muscle weakness (n = 8). NS: not significant.
(DOCX)
