# Supplementary figures and images for: Investigation of Fatigability during Repetitive Robot-Mediated Arm Training in People with Multiple Sclerosis
Source: PLoS One. 2015 Jul 27;10(7):e0133729. doi: 10.1371/journal.pone.0133729 (PMC4516328; doi:10.1371/journal.pone.0133729)

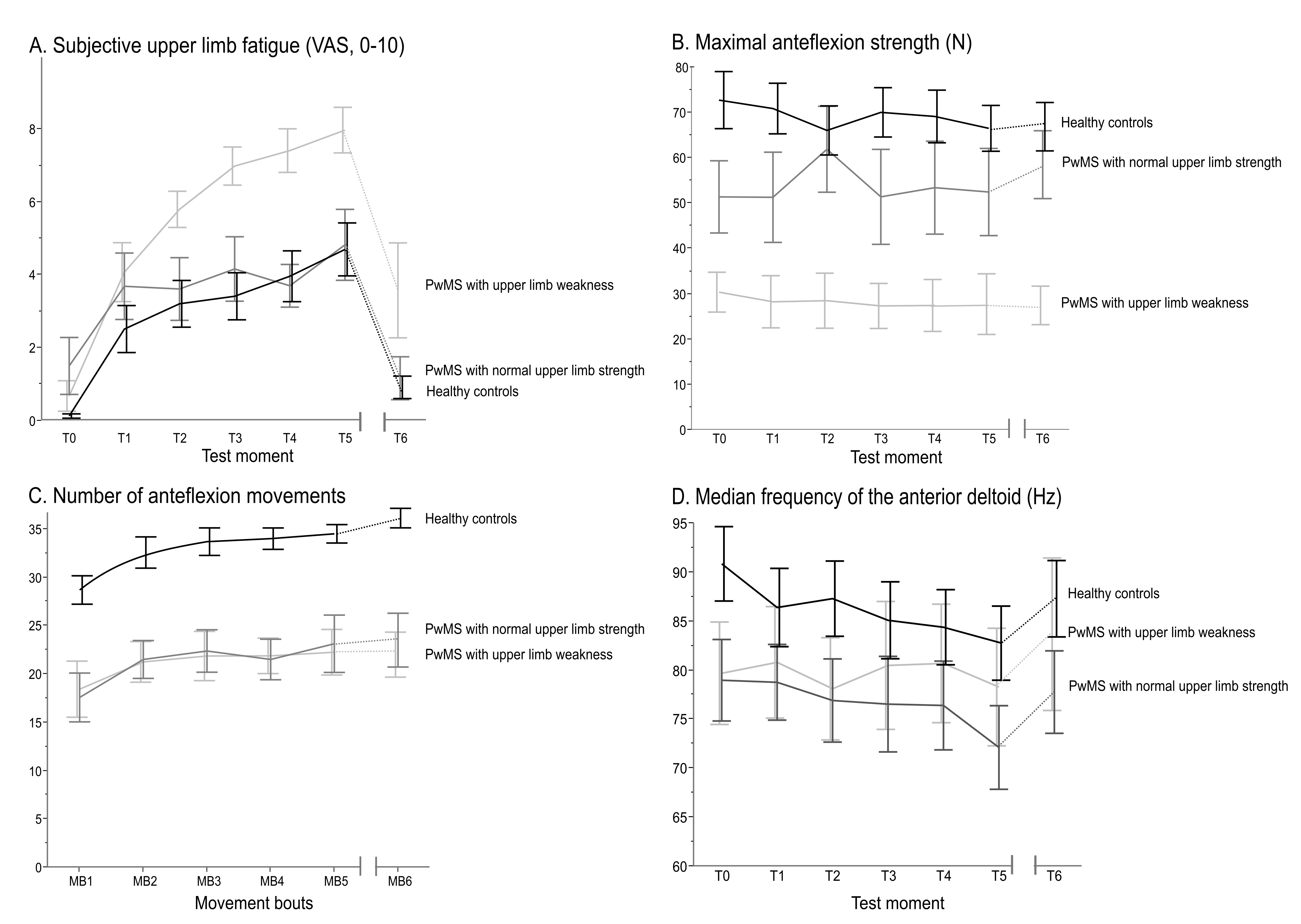

Supplement: S1 Fig — PwMS: people with multiple sclerosis. (A) The score for the perceived fatigue in the arm. (B) The maximal anteflexion strength. (C) The number of anteflexion movements within each movement bout of three minutes. (D) Median frequency of the anterior deltoid. (TIF) [file pone.0133729.s001.tif]
