# Supplementary material for: Investigation of Fatigability during Repetitive Robot-Mediated Arm Training in People with Multiple Sclerosis
Source: PLoS One. 2015 Jul 27;10(7):e0133729. doi: 10.1371/journal.pone.0133729 (PMC4516328; doi:10.1371/journal.pone.0133729)
Supplement: S1 Table — PwMS: people with multiple sclerosis. (DOCX) [file pone.0133729.s002.docx]

|  | | | | | | | | | | | |
| --- | --- | --- | --- | --- | --- | --- | --- | --- | --- | --- | --- |
|  |  |  | Time of testing | | | | | | | | |
|  |  |  | T0 | T1 | T2 | T3 | T4 | T5 |  | T6 | T7 |
| **Maximal anteflexion strenght** | Healthy controls | Mean | 72.76 | 70.90 | 66.07 | 70.05 | 69.15 | 66.53 | Rest | 66.90 | 66.28 |
|  |  | SD | 25.21 | 22.35 | 20.97 | 21.09 | 23.24 | 20.23 |  | 21.36 | 21.60 |
|  | PwMS | Mean | 40.18 | 39.78 | 43.92 | 39.37 | 40.44 | 40.11 |  | 41.95 | 39.51 |
|  |  | SD | 19.64 | 25.21 | 25.58 | 25.63 | 26.36 | 24.67 |  | 22.31 | 27.33 |
|  |  |  |  |  |  |  |  |  |  |  |  |
| **VAS score** | Healthy controls | Mean | 0.13 | 2.51 | 3.21 | 3.41 | 3.96 | 4.70 |  | 0.91 | 3.08 |
|  |  | SD | 0.24 | 2.58 | 2.57 | 2.59 | 2.79 | 2.91 |  | 1.23 | 2.42 |
|  | PwMS | Mean | 1.09 | 3.88 | 4.71 | 5.58 | 5.68 | 6.40 |  | 2.37 | 4.86 |
|  |  | SD | 1.77 | 2.36 | 2.23 | 2.46 | 2.48 | 2.77 |  | 3.03 | 3.41 |
|  |  |  |  |  |  |  |  |  |  |  |  |
| **RMS of the anterior deltoid (microvolts)** | Healthy controls | Mean | 51.26 | 47.74 | 52.84 | 54.49 | 55.03 | 56.64 |  | 60.62 | 56.89 |
|  |  | SD | 15.41 | 13.74 | 18.91 | 18.46 | 16.22 | 15.99 |  | 15.28 | 19.10 |
|  | PwMS | Mean | 44.95 | 41.90 | 43.24 | 44.98 | 45.68 | 42.23 |  | 43.14 | 45.63 |
|  |  | SD | 25.12 | 23.52 | 25.25 | 25.94 | 26.57 | 23.86 |  | 23.90 | 26.71 |
|  |  |  |  |  |  |  |  |  |  |  |  |
| **MDF of the anterior deltoid (microvolts)** | Healthy controls | Mean | 90.85 | 86.40 | 87.30 | 85.09 | 84.39 | 82.76 |  | 87.30 | 82.08 |
|  |  | SD | 15.17 | 15.97 | 15.36 | 15.70 | 15.34 | 15.13 |  | 15.59 | 14.70 |
|  | PwMS | Mean | 79.34 | 79.79 | 77.51 | 78.51 | 78.55 | 75.20 |  | 80.53 | 74.84 |
|  |  | SD | 12.91 | 13.32 | 13.01 | 15.87 | 14.76 | 14.57 |  | 16.17 | 17.67 |
