# Supplementary material for: Investigation of Fatigability during Repetitive Robot-Mediated Arm Training in People with Multiple Sclerosis
Source: PLoS One. 2015 Jul 27;10(7):e0133729. doi: 10.1371/journal.pone.0133729 (PMC4516328; doi:10.1371/journal.pone.0133729)
Supplement: S2 Table — PwMS: people with multiple sclerosis. (DOCX) [file pone.0133729.s003.docx]

|  | | | | | | | | | |
| --- | --- | --- | --- | --- | --- | --- | --- | --- | --- |
|  |  |  | Exercise number | | | | | | |
|  |  |  | Ex1 | Ex2 | Ex3 | Ex4 | Ex5 |  | Ex6 |
| **Number of anteflexion movements** | Healthy controls | Mean | 28.69 | 32.56 | 33.69 | 34 | 34.5 | Rest | 36.13 |
|  |  | SD | 5.88 | 6.46 | 5.69 | 4.43 | 3.81 |  | 4.05 |
|  | PwMS | Mean | 18 | 21.38 | 22.13 | 21.69 | 22.69 |  | 22.75 |
|  |  | SD | 6.93 | 5.55 | 6.23 | 5.39 | 7.34 |  | 7.06 |
|  |  |  |  |  |  |  |  |  |  |
| **Average time per anteflexion movement (seconds)** | Healthy controls | Mean | 6.55 | 5.68 | 5.43 | 5.3 | 5.17 |  | 4.98 |
|  |  | SD | 2.07 | 1.39 | 1.05 | 0.66 | 0.52 |  | 0.54 |
|  | PwMS | Mean | 10.73 | 8.85 | 8.5 | 8.48 | 8.53 |  | 8.52 |
|  |  | SD | 3.84 | 2.72 | 2.46 | 2.44 | 2.86 |  | 2.87 |
|  |  |  |  |  |  |  |  |  |  |
| **Average trajectory per anteflexion movement (metres)** | Healthy controls | Mean | 0.93 | 0.9 | 0.89 | 0.88 | 0.87 |  | 0.86 |
|  |  | SD | 0.13 | 0.12 | 0.07 | 0.05 | 0.05 |  | 0.05 |
|  | PwMS | Mean | 1.12 | 0.98 | 0.99 | 0.99 | 0.96 |  | 0.97 |
|  |  | SD | 0.3 | 0.17 | 0.16 | 0.16 | 0.16 |  | 0.16 |
