# Supplementary material for: Investigation of Fatigability during Repetitive Robot-Mediated Arm Training in People with Multiple Sclerosis
Source: PLoS One. 2015 Jul 27;10(7):e0133729. doi: 10.1371/journal.pone.0133729 (PMC4516328; doi:10.1371/journal.pone.0133729)
Supplement: S3 Table — NS: not significant. (DOCX) [file pone.0133729.s004.docx]

|  | | | |
| --- | --- | --- | --- |
| Variable | Main effect of group | Main effect of time | Group*time interaction |
|  |  |  |  |
| Subjective fatigue feelings (VAS) | NS | p<0.001 | p<0.0001 |
| Maximal anteflexion strength (N) | p<0.001 | NS | NS |
| RMS anterior deltoid (Microvolts) | NS | NS (0.08) | p<0.05 |
| MDF anterior deltoid (Hz) | NS | p<0.0001 | p<0.05 |
|  |  |  |  |
| Number of movements | p<0.001 | p<0.0001 | NS |
| Average movement trajectory (m) | p<0.01 | p<0.0001 | NS |
| Average movement time (s) | p<0.01 | p<0.0001 | NS |
|  |  |  |  |
|  |  |  |  |
